# Compensatory effects of *M. tuberculosis rpoB* mutations outside the rifampicin resistance-determining region

**DOI:** 10.1080/22221751.2021.1908096

**Published:** 2021-03-29

**Authors:** Pengjiao Ma, Tao Luo, Liang Ge, Zonghai Chen, Xinyan Wang, Rongchuan Zhao, Wei Liao, Lang Bao

**Affiliations:** Laboratory of Infection and Immunity, West China School of Basic Medical Sciences & Forensic Medicine, Sichuan University, Chengdu, People’s Republic of China

**Keywords:** *M. tuberculosis*, rifampicin resistance, *rpoB* mutation, fitness cost, fitness compensation

## Abstract

*Mycobacterium tuberculosis* has been observed to develop resistance to the frontline anti-tuberculosis drug rifampicin, primarily through mutations in the rifampicin resistance-determining region (RRDR) of *rpoB*. While these mutations have been determined to confer a fitness cost, compensatory mutations in *rpoA* and *rpoC* that may enhance the fitness of resistant strains have been demonstrated. Recent genomic studies identified several *rpoB* non-RRDR mutations that co-occurred with RRDR mutations in clinical isolates without *rpoA/rpoC* mutations and may confer fitness compensation. In this study, we identified 33 evolutionarily convergent *rpoB* non-RRDR mutations through phylogenomic analysis of public genomic data for clinical *M. tuberculosis* isolates. We found that none of these mutations, except V170F and I491F, can cause rifampin resistance in *Mycolicibacterium smegmatis*. The compensatory effects of five representative mutations across *rpoB* were evaluated by an *in vitro* competition assay, through which we observed that each of these mutations can significantly improve the relative fitness of the initial S450L mutant (0.97–1.08 vs 0.87). Furthermore, we observed that the decreased RNAP transcription efficiency introduced by S450L was significantly alleviated by each of the five mutations. Structural analysis indicated that the fitness compensation observed for the non-RRDR mutations might be achieved by modification of the RpoB active centre or by changes in interactions between RNAP subunits. Our results provide experimental evidence supporting that compensatory effects are exerted by several *rpoB* non-RRDR mutations, which could be utilized as additional molecular markers for predicting the fitness of clinical rifampin-resistant *M. tuberculosis* strains.

## Introduction

Tuberculosis (TB) remains the world’s leading cause of death from an infectious agent, namely, *Mycobacterium tuberculosis* (MTB). According to the World Health Organization, there were approximately 10 million new cases and 1.4 million deaths due to MTB infection in 2019, and the best estimate was 465,000 incident cases of rifampicin-resistant TB (RR-TB); 78% of these cases were multidrug-resistant TB (MDR-TB, which is resistant to both isoniazid and rifampicin) [1]. The currently recommended treatment regimen for drug-susceptible TB cases is a 6-month combination therapy of four first-line drugs: isoniazid, rifampicin, ethambutol and pyrazinamide, however, in 2019, the success rate for this regimen was only 85% [1]. Unfortunately, the treatment of MDR-TB requires second-line drugs that are more expensive and toxic but less effective than first-line drugs and exhibit a lower success rate (57%) [1–4]. In 2019, 206,030 patients with MDR-TB were reported worldwide, and approximately 6% of these patients developed XDR-TB (MDR-TB plus resistance to a fluoroquinolone and an injectable agent); clearly, drug resistance is a major obstacle to global TB control [1]. Mathematical models have predicted that because of the fitness cost associated with drug-resistant mutations that impair the reproduction or virulence of MDR/XDR-TB strains, it is difficult for the bacteria to maintain large transmission networks or concentrated outbreaks [5,6]. However, molecular epidemiology has demonstrated that the spread of MDR/XDR-TB still occurs frequently [7,8]. The acquisition of compensatory mutations appears to play a role in epidemics; that is, drug-resistant bacteria can enhance or even restore their fitness through compensatory evolution, which may promote the competitive advantage of bacteria in the population and facilitate their spread [9–11].

RIF is one of the most effective first-line anti-TB drugs, it acts as a bactericidal agent by binding to the β subunit of RNA polymerase (RNAP) and preventing the extension of the primary RNA product, which ultimately inhibits transcription [12,13]. Due to the lack of continuing horizontal gene transfer, a large majority of drug resistance phenotypes in *M. tuberculosis* are caused by chromosomal mutations, and RIF resistance is no exception [4]. *M. tuberculosis* gains rifampicin resistance primarily through *rpoB* mutations, and more than 95% of these mutations are present within an 81-bp rifampicin resistance-determining region (RRDR, corresponding to codons 426–452 in *M. tuberculosis* and corresponding to codons 507–533 in *E. coli*) [14]. Mutations in codons 450, 445 and 435 are common among clinical rifampicin-resistant isolates; of these mutations, S450L occurs most frequently [15,16]. Mutations in *rpoB* RRDR result in alterations to the structure of the RIF-binding pocket and confer rifampicin resistance by decreasing the binding affinity of RIF to RNAP [12]. In addition to resistance, *rpoB* mutations confer bacterial fitness costs by either directly decreasing the transcriptional efficacy of RNAP or indirectly altering genome-wide transcriptional profiles [17,18].

The existence of fitness cost due to RRDR mutations could result in impaired growth rate to the resistant strains; however, a previous study demonstrated that clinically derived mutant *M. tuberculosis* strains often had a significantly higher fitness than laboratory-derived mutants [19], one of the possible explanations is that clinical strains accumulated compensatory mutations [18,20,21]. Iñaki Comas et al. reported that secondary mutations in *rpoA* or *rpoC* (encoding α and β′ subunits of the polymerase) can alleviate the fitness cost of initial *rpoB* mutations, and more than 30% of MDR clinical isolates had such a mutation, indicating that compensatory evolution plays a role in the transmission of MDR-TB [9]. Although discrepancies have been reported [22], it has been generally observed that compensatory mutations in *rpoA* and *rpoC* have facilitated the spread of drug-resistant *M. tuberculosis* during past several decades [10,11]. In addition to *rpoA* and *rpoC*, several previous studies showed that secondary mutations in *rpoB* itself also have compensatory functions in bacteria, including *E. coli*, *P. aeruginosa* and *S. enterica,* through an *in vitro* experimental evolution assay [23–26]. In *M. tuberculosis*, a recent study demonstrated that the *rpoB* V534M mutation occurred in a clinical outbreak strain and functioned as a compensatory mutation to ameliorate the fitness cost introduced by the primary mutation S450L [27]. Furthermore, several genome sequencing-based studies demonstrated that many rifampicin-resistant *M. tuberculosis* clinical isolates carry double/multiple *rpoB* mutations, with nonsynonymous mutations outside the RRDR commonly co-occurring with RRDR mutations and being more likely to appear in strains without *rpoA* or *rpoC* mutations [2,8,28], which suggests that these mutations may have a compensatory function.

To investigate the function of *rpoB* non-RRDR mutations occurring in clinical *M. tuberculosis* isolates, we systematically analysed *rpoB* mutations by examining published whole genome sequencing data for globally collected MTB strains, through which we identified numerous convergent mutations outside the RRDR. By using *Mycolicibacterium smegmatis* as a model organism, we tested the potential of a single mutation to cause rifampicin resistance, confirmed the compensatory functions of five non-RRDR mutations through an *in vitro* competitive growth assay, and further investigated the effects of these mutations on restoring the activity of RNAP. The compensatory mechanisms underlying the effects of the mutations on RNAP were elucidated by analysing their potential modifications to the structure of the RNAP.

## Materials and methods

### Bioinformatic analysis

Short read data from previous projects (PRJEB2138, PRJNA282721, PRJNA187550 and PRJNA283583) were downloaded from the NCBI SRA database. The reads were first trimmed using Fastp (v0.20.0, https://github.com/OpenGene/fastp) and were subsequently mapped to the reference genome of H37Rv (GenBank, AL123456) with Bowtie2 (v2.4.1, http://bowtie-bio.sourceforge.net/bowtie2) in end-to-end mode. Reads that mapped to more than one genomic position were discarded. GATK (v3.8, https://gatk.broadinstitute.org) and Bcftools (v1.9, https://gatk.broadinstitute.org) were employed to call and filter genomic single nucleotide variants (SNVs), which were subsequently annotated by SnpEff (v4.3 T, http://snpeff.sourceforge.net). Genomes with nonsynonymous *rpoB* SNVs outside the RRDR were selected, and a maximum likelihood (ML) phylogeny was constructed based on genomic SNVs outside the PE/PPE and PE-PGRS genes using RaxML (v8.2.12, https://cme.h-its.org/exelixis/web/software/raxml/). SNVs in *rpoB* were mapped to the phylogeny assuming parsimonious acquisition with no reversion.

### Point mutagenesis and resistance screening

*M. smegmatis* was transformed with a modified pJV53 plasmid (constructed in this study), which carrying the mycobacteriophage recombinases Gp60 and Gp61, the kanamycin resistance gene (*kanR*), the mutated hygromycin resistance gene (*hygR*) with two adjacent nonsense mutations that inactivate its function and the counterselectable gene *sacB* [Supplementary Figure S3]. The bacteria were grown to the logarithmic phase, induced with 0.2% acetamide (Sangon Biotech, Shanghai) for 5 h and electrocompeted as described previously [29]. For resistance screening, oligos carrying individual *rpoB* mutations [Supplementary Table S1] were mixed with the *hygR*-restoring oligo in a ratio of 5:1 and electroporated into 200 μl electrocompetent cells. For *rpoB* mutant screening, oligos carrying individual *rpoB* mutations were electroporated either individually or in combination with Oligo-S450L (at a ratio of 1:1) into 200 μl electrocompetent cells. Electroporation was performed with 2 μl (10 μM) oligos, 2.5 kV, and 25 μF 1000 Ω. bacterial suspensions were recovered at 37°C for 4 h in Middlebrook 7H9 (Difco, USA) and subsequently plated onto Middlebrook 7H10 (Difco, USA) containing 50 μg/ml RIF (Sangon Biotech, Shanghai) and/or 100 μg/ml Hyg for 3–5 days at 37°C. The numbers of resistant colonies in each plate were counted. For RIF-containing plates that showed a high resistance rate, 5∼10 colonies were selected for PCR amplification and DNA sequencing (primers are listed in Supplementary Table S2) to determine *rpoB* mutations. Mutations codons were numbered according to the numbering system in *M. tuberculosis* H37Rv proposed in 2002 [30]. Resistant colonies with single or double *rpoB* mutations (one colony for each genotype) were collected for serial passaging for four generations and were subsequently plated on 15% sucrose plates to select colonies that lost the pJV53 plasmid.

### Bacterial culture and MIC analysis

*M. smegmatis* was grown in nutritious Middlebrook 7H9 supplemented with 0.5% (v/v) glycerol (Sangon Biotech, Shanghai), 0.05% (v/v) Tween-80 (Sangon Biotech, Shanghai) and 10% (v/v) ADC. MICs were quantified with the resazurin microdilution titration method. Briefly, Middlebrook 7H9 broth was added to 96-well plates to prepare a continuous two fold dilution of rifampicin in triplicate. Each well was inoculated with 10^5^ CFU bacteria for 2–3 days at 37 °C and subsequently mixed with 30 μl 0.01% resazurin (Sangon Biotech, Shanghai) solution to determine the lowest concentration that inhibited bacterial growth. Three different gradients of rifampicin were tested: 600, 300, 150, 75, 37.5, and 18.75 μg/ml; 500, 250, 125, 62.5, 31.25, and 15.625 μg/ml; and 400, 200, 100, 50, 25, and 12.5 μg/ml.

## *In vitro* competition assay

Mutant strains competed against the wild-type strain in pairs in nutritionally deficient 7H9 medium containing 0.02% glycerol as the sole carbon source. Briefly, *M. smegmatis* strains were grown in regular Middlebrook 7H9 until logarithmic phase, and then the culture was washed three times and resuspended in nutritionally deficient 7H9 broth. The single cell suspension (OD_600_≈0.02) of each mutant strain was mixed with the wild-type strain in a 1:1 ratio, subsequently plated on selective (containing 50 μg/ml RIF) and nonselective (containing no drug) 7H10 agar for baseline CFU counts. For competitive growth, the 1:1 mixture was inoculated into 10 ml of nutritionally deficient 7H9 medium. After 72 h, the cultures were plated on both selective and nonselective 7H10 agar to obtain endpoint CFU counts. The relative competitive fitness was calculated following the formula previously described [31]. For the experiment, three independent replicates were performed for each mutant.

### Transcription efficiency assay

Each strain was inoculated in nutritionally deficient 7H9 medium to logarithmic phase, and the culture was subsequently washed three times with PBS containing 0.05% Tween 80. Next, 2 mg bacterial cells was retained for basal expression analysis, and the remaining cells were incubated in prewarmed 7H9 broth (containing 2 mg/ml acetamide as the sole carbon source) at 37°C to induce the acetamidase gene *amiE*. Bacterial cells were harvested after induction for 20, 40, 60 and 90 min. Total RNA was extracted and reverse transcribed to cDNA, and real-time PCR was performed using TB Green^TM^ Premix Ex TaqTM II kit (TaKaRa) on the CFX Connect (Bio-Rad) with primers listed in Supplementary Table S3. The expression of *amiE* relative to the constitutive gene *sigA* was calculated through the 2^−ΔΔCT^ method. The transcription efficiency was defined as the slope of the regression line by plotting the proportional change in 2^-ΔΔCT^ over time [32].

### Structure analysis of RNAP

The crystal structure of *M. tuberculosis* RNAP in complex with rifampin (PDB: 5UHB) were employed to analyse the potential resistance or compensatory mechanism of *rpoB* mutations. The RNAP subunits were depicted, and the mutant residues were located with PyMOL v2.3.1 (https://pymol.org).

### Statistical analysis

Statistical differences between *M. smegmatis* strains in fitness and transcriptional efficiency were analysed using unpaired Student's t-test by GraphPad Prism (v7.0). *P* values < 0.05 were considered statistically significant.

## Result

### Identification of convergent rpoB mutations outside RRDR

**Figure 1.**
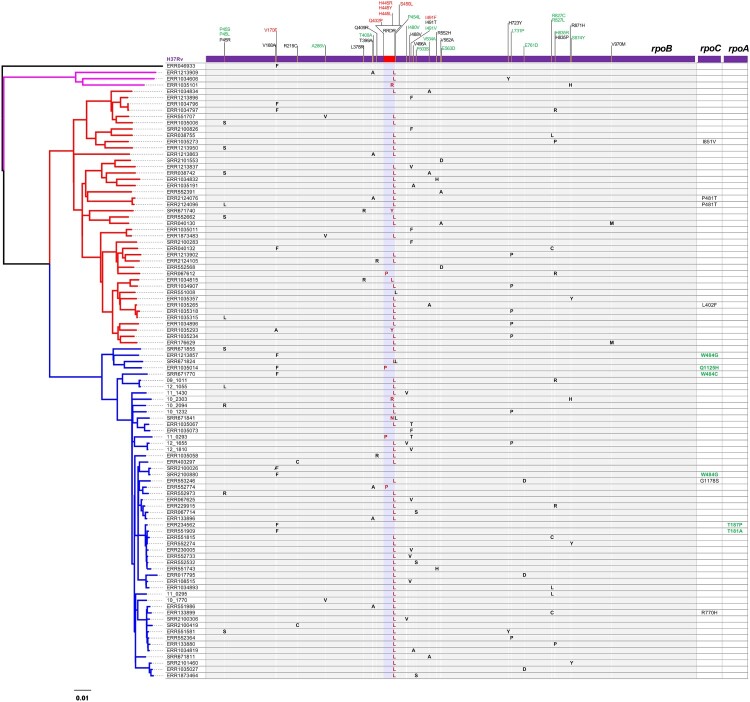
Phylogenomic analysis of convergent *rpoB* non-RRDR mutations. Left, maximum likelihood of 98 *M. tuberculosis* isolates. Right, mutation profile of the *rpoABC* operon. The RRDR region and resistance-conferring mutations are highlighted in red. Convergent mutations independently emerging at least three times are in green. Compensatory *rpoA/C* mutations reported previously are highlighted in green.

A total of 504 isolates that carry 304 different *rpoB* mutations outside the RRDR were identified through mutation analyses from genome sequencing data of 6,772 *M. tuberculosis* isolates worldwide. By mapping the mutations onto the ML phylogeny, we identified 33 convergent mutations that independently emerged at least twice in 263 isolates. By keeping only one isolate from each monophyletic clade that contained strains with identical mutation profiles in the *rpoABC* operon, a final set of 98 strains was selected, and an ML phylogeny was constructed based on genomic SNVs [Figure 1]. According to the mutation profiles, 80 isolates were determined to harbour mutations in the RRDR. There were 30 different non-RRDR mutations identified among the 80 isolates, and except for mutation P454L, all the remaining 29 mutations distributed in 78 strains solely co-occurred with RRDR mutations. Among the 29 mutations, 23 distributed among 70 strains exclusively co-occurred with S450L. For the remaining six mutations, R871H and L378R exclusively co-occurred with mutation H445R/Y/L, H835R co-occurred with S450L, V170F or Q432P, I491 T co-occurred with S450L and Q432P, V168A co-occurred with H445Y, and T400A co-occurred with S450L and Q432P. No compensatory mutation in *rpoA* or *rpoC* was identified in any of the 80 strains. For the 18 strains with only non-RRDR mutations, 10 strains harboured V170F, five strains harboured I491F, two strains harboured E563D and one strain harboured P454L. Mutations V170F and I491F have been proven to confer rifampicin resistance [33,34], while the role of E563D and P454L in rifampicin resistance has not been elucidated. The P454L mutation was also identified to co-occur with S450L and H445N in the two strains. Six strains with the single mutation V170F in *rpoB* were determined to harbour potential compensatory mutations in *rpoC* and *rpoA* [Figure 1].

### Effects of non-RRDR mutations on RIF resistance

First, we investigated whether convergent mutations outside RRDR confer rifampicin resistance. Sequence alignment showed that the amino acid residues in the convergent sites were all conserved between *M. tuberculosis* and *M. smegmatis* [Supplementary Figure S2]. Therefore, we chose *M. smegmatis t*o further study the function of individual mutations. We first tested the potential to cause rifampicin resistance for the 22 mutations that independently emerged at least three times according to the phylogeny. Each of the oligos carrying individual mutations was transformed together with the *hygR*-restoring oligo into Che9c Gp60/61-expressing *M. smegmatis.* The growth of numerous colonies in the hygromycin containing plates indicates success of the transformation and homologous recombination for all groups [Supplementary figure S1b]. Since the homologous recombination rate is considerably higher than the spontaneous mutation rate [35], if a *rpoB* mutation confers RIF-resistance, the corresponding transformed group should obtain a considerably higher resistance rate than the control (with no oligo transformation) on the rifampicin containing plates . This notion was confirmed by transforming oligos that contain the well-known resistance conferring mutation S450L, which was used as a positive control [Supplementary Figure S1a]. For the remaining 21 oligos carrying different non-RRDR mutations, only two of them, namely, Oligo-I491F and Oligo-V170F, were found to significantly increase the resistance rate in plates containing 50 μg/ml RIF [Supplementary Figure S1a]. PCR amplification and DNA sequencing were performed on 5∼10 single colonies in each of these two plates, and the results confirmed that all the colonies in each plate carried the specific mutation corresponding to the transformed oligo. The MIC results of the I491F and V170F mutant strains were 100 and 125 μg/ml, respectively, which is consistent with the findings obtained in previous studies [33,36] [Table 1].

**Table 1.** Rifampicin MICs for strains of *M. smegmatis mc^2^155.*

Strains	MIC (μg/ml)
Wild-type	18.75
*rpoB* I491V	18.75
*rpoB* V170F	125
*rpoB* I491F	100
*rpoB* S450L	300
*rpoB* S450L + P45L	300
*rpoB* S450L + P45S	300
*rpoB* S450L + I480V	300
*rpoB* S450L + V534M	300
*rpoB* S450L + R827L	300

Since most of the non-RRDR mutations exclusively co-occurred with S450L, we further studied whether these mutations could change the rifampicin resistance level of the primary S450L mutant. Five mutations, P45L, P45S, I480 V, V534M and R827L, which are distributed in different regions of *rpoB,* were selected for the test. Oligos (Oligo-P45L, Oligo-P45S, Oligo-I480 V, Oligo-V534M and Oligo-R827L) carrying the above mutations were individually mixed with Oligo-S450L and subsequently transformed into Che9c Gp60/61-expressing *M. smegmatis*. Resistance mutants were screened on RIF (50 μg/ml)-containing plates. For each pair of transformations, colonies with single S450L mutations or double mutations were obtained. Again, no colony with a single non-RRDR mutation was identified. The MICs of the double mutant strains were all the same as those of the S450L single mutant [Table 1]. Taken together, the results described above suggest that the convergent mutations that exclusively co-occurred with RRDR mutations could not result in rifampicin resistance and were more likely to confer fitness compensation.

### Effects of non-RRDR mutations on fitness compensation

**Figure 2.**
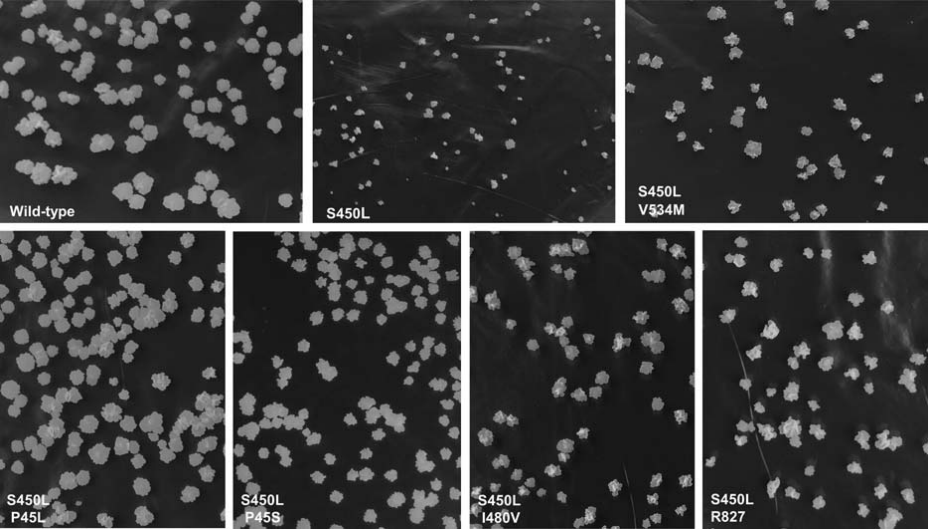
Colony morphology of wild-type and mutant *M. smegmatis* strains after growing on 7H10 plates for 4 days.

To examine the compensation effects of non-RRDR mutations, the growth rate and relative fitness of the S450L single mutant strain and the five double mutant strains were analysed in solid and liquid culture, respectively, compared to the wild-type strain. In the 7H10 solid plates, the growth defect of the S450L single mutant strain was clear compared with the wild-type strain. The growth rates of the double mutant strains were apparently higher than the S450L single mutant, suggesting that secondary mutations P45L, P45S, I480 V, V534M and R827L could compensate for the growth defects introduced by mutation S450L to varying degrees [Figure 2]. Notably, the P45L mutation almost fully compensated for the growth defect induced by S450L.

**Figure 3.**
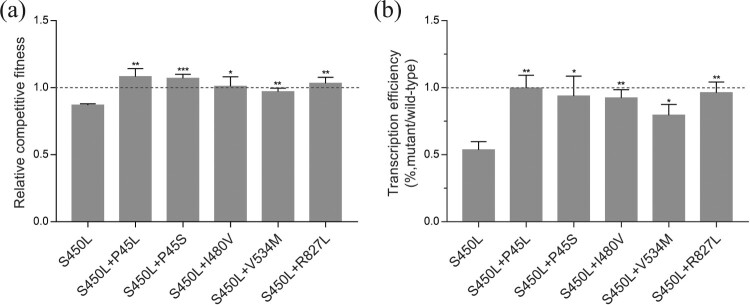
(a) *In vitro* competitive fitness of the *rpoB* mutant strains in nutritionally deficient 7H9 medium. (b) Relative transcription efficiency of the mutant strains compared to the wild-type strain. The data are shown as the mean ± SD from three independent experiments. To measure the compensation effect of double mutation, the statistical differences of double mutant strains versus the S450L single mutant strain were analysed using unpaired Student's t-test, *P < 0.05, **P < 0.01, ***P < 0.001 (*t* = 3.036∼10.95, *df* = 4).

The growth rate of the S450L single mutant strain in 7H9 broth-rich liquid medium was comparable to that of the wild-type strain (data not shown). Therefore, we attempted to culture the strains under different nutrient starvation conditions, as suggested by a previous study[32], and we found that under conditions with a low concentration of glycerol (0.02%) as the sole carbon source, the S450L single mutant strain showed significant growth defects compared to the wild-type strain. Under this nutrient-limited condition, we applied the pairwise competition assay to quantify the relative fitness of the single and double mutant strains compared to the wild-type strain. The S450L single mutant strain showed a significant decrease in fitness, exhibiting a relative fitness of 0.87 (95% CI, 0.85–0.89). The relative fitness of all five double mutant strains was significantly higher than that of the single mutant [Figure 3(a)]. The V534M mutation was included as a positive control in our experiments, and the compensatory effect observed in this study is consistent with the findings of a previous study in BCG [27]. Based on the above mentioned results, we conclude that *rpoB* non-RRDR mutations could compensate for the growth defects caused by S450L.

### Restoration of RNAP activity by non-RRDR mutations

According to recent studies, the fitness cost of rifampicin-resistance mutations in *rpoB* could be attributable to the reduction in the transcriptional efficiency of RNAP [18,37]. A quantitative real-time PCR assay described previously was applied to evaluate the RNAP activity of the wild-type, single-mutant and double-mutant strains [32]. According to the assay, the expression of the inducible acetamidase gene *amiE* relative to the gene *sigA* was measured by real-time PCR, and the transcription efficiency was defined as the kinetics of the *amiE* transcript relative to the constitutive *sigA* transcript. Our results showed that the transcription efficiency of the single mutant strain was approximately 54% (95% CI, 0.42–0.63) of the wild-type strain. The transcription efficiency of the double mutant strains were all significantly higher than those of the single mutant, exhibiting an efficiency at least 80% of the wild type or even comparable to those of the wild type [Figure 3(b)].

## Discussion

In the current study, we identified 33 *rpoB* non-RRDR mutations that are under potential selection in clinical isolates. By excluding the potential to cause rifampicin resistance, our results suggest that mutations that exclusively co-occurred with RRDR mutations likely represent compensatory mutations. The compensatory effects of five representative mutations were further confirmed by finding their ability to alleviate growth defects and to enhance the transcriptional efficiency of RNAP in the parent strain. Our results support the previous finding that the fitness cost of rifampicin-resistance mutations was attributable to their direct influence on RNAP activity [38]. Our data further indicate that compensatory mutations could enhance the fitness of rifampicin-resistant strains by restoring the enzymatic activity of RNAP.

**Figure 4.**
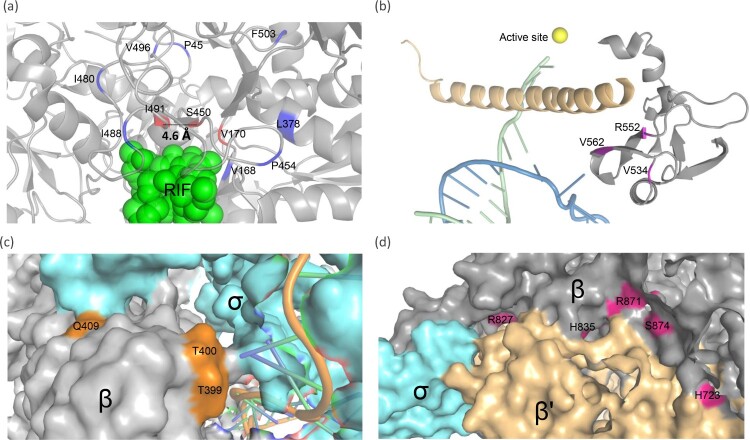
Structure modelling of RNAP and potential compensatory mechanisms of *rpoB* non-RRDR mutations. (a) Mutations spatially around the RRDR. Potential compensatory loci are coloured in blue, and rifampicin-resistant loci are shown in red. β subunit, grey; β’ subunit, yellow; σ factor, light blue. (b) Mutations located in the region interacting with the bridge helix of the β′ subunit. The template and nontemplate DNA strands are shown in green and blue, respectively. The Mg^2+^ ion in the active centre is shown by a gold sphere. β and β’ subunits are shown as cartoons in grey and yellow. (c) Mutations located in the interaction surface with the σ factor in the upstream edge of the nontemplate DNA channel. (d) Mutations located at the interface between the β and β’ subunits.

The recently obtained high-resolution structure of *M. tuberculosis* RNAP enables us to derive possible explanations for the compensation mechanism of the non-RRDR mutations [39]. As described, RRDR mutations lead to structural or surface electrostatic potential change in the catalytic centre [39], which may reduce transcriptional efficiency and bacterial fitness [27,38,40]. The non-RRDR mutations could be classified into two groups according to their spatial distance to the RRDR. The first group contains mutations that are spatially adjacent to the RRDR or specifically the S450 residue [Figure 4(a)]. Notably, most of these mutations are located in loop regions. Loops are the most flexible parts of a protein, and mutations in the loop regions could contribute to modulation or diversification of protein functions [41–43]. For RNAP, the rearrangement of the loop region near the active site plays an important role in its transcriptional activity [42]. Accordingly, we surmise that secondary mutations around the RRDR may modulate the structure and/or electrostatic distributions of the active centre, which could enhance the transcriptional activity of *rpoB* mutants. Notably, three mutations with resistance or compensatory function were found in the same codon 491, which has a direct Van der Waals force with rifampin [40–44]. Compared to wild-type isoleucine (Ile, I), which has a simple CH_3_ chain, the resistance conferring phenylalanine (Phe, F) residues contains a bulky benzyl side chain, which may cause a steric conflict and prevent the binding of rifampicin. The remaining two mutant residues, threonine (Thr, T) and valine (Val, V) have shorter side chains than isoleucine, thus, these residues could have no or minor influence on the binding affinity of RIF. Indeed, we have obtained the strain caring single I491 V mutation and found its MIC to RIF have no difference with the wild-type [Table 1]. As these two mutations exclusively co-occur with S450L and codon 491 is spatially close to codon 450 (4.6 Å) [Figure 4(a)], we speculate that they most likely function as compensatory mutations by counteracting the structural/electrostatic changes introduced by S450L.

The second group contains mutations that are spatially distant from the rifampicin binding pocket, and most of them are located at the interface between subunits [Figure 4]. Mutations V534A, R552A and V562A were mapped to a region that interacts with the bridge helix of the β′ subunit [Figure 4(b)], which is an important structure for moving and positioning the DNA template strand into the active centre. These mutations may influence the dynamic properties of increasing the elongation rate of the bridge helix, which has been previously demonstrated in *S. enterica* [26,45].

Mutations L378R, T399A, T400A and Q409R were located in the interaction surface with the σ factor at the upstream edge of the “transcription bubble” [Figure 4(c)]. The amino acid residuals in this region are important for the formation and maintenance of bubbles, which are necessary for the formation of the RNAP-DNA open complex [39,46–48]. We propose that the above mutations could promote transcription initiation, thereby enhancing transcription efficiency. Mutations H723Y, R827C/L, H835P/R, R871H and S874Y are located at the region interfacing the β′ subunit [Figure 4(d)]. These mutations are distant from any functional domain, and their compensatory mechanism remains to be elucidated.

Most of the potential compensatory mutations (70/78, 89.7%) outside the RRDR co-occurred with S450L. The dominance of S450L in strains with compensatory mutations in *rpoC* and *rpoA* has also been observed in previous studies [2,10,49]. It has been proven that the S450L mutation is associated with high-level rifampicin resistance and causes a small fitness defect compared to other *rpoB* mutations [19,50,51], which may enable it to outcompete other *rpoB* mutations during the emergence of resistance within the host and contribute to its initial dominance among clinical rifampicin-resistant isolates [52,53]. The relatively high fitness of S450L mutant strains could also facilitate their transmission within the human population [37]. Since the emergence of compensatory mutations is time-dependent [9], the greater persistence of S450L mutant strains among the human population due to their more successful transmissions may endow them with a greater chance of further evolving compensatory mutations which, in turn, may further facilitate their transmission and contribute to their final dominance.

Four non-RRDR mutations were determined to be unlinked with RRDR mutations, and two of them (V170F and I491F) were proven to confer rifampicin resistance, which is consistent with previous studies [34,54,55]. Mutations in codon 454 (P454L, P454S, P454R and P454 T) have been previously identified in rifampicin-resistant strains, and in most cases, they co-occur with other RRDR mutations [56,57]. In the current study, P454L was found in 3 strains, and one of them did not have any RRDR mutation, suggesting a potential association with rifampicin resistance. However, a clinical *M. tuberculosis* isolate with the P454L mutation was found with an MIC < 0.25 μg/ml in a recent study [56]. Considering that the genetic background of the clinical strain could result in heterogeneity of resistance levels, it is possible that P454L may lead to low levels of rifampicin resistance in a few clinical strains. The effect of the remaining E563D mutation on rifampicin resistance has not been evaluated previously, and our results in *M. smegmatis* indicate that this mutation has no effect on rifampicin resistance. This finding might be attributable to the structural difference of *rpoB* between *M. tuberculosis* and *M. smegmatis*, i.e. the residues around codon 563 are more variable than those flanking codons 170 and 491 [Supplementary Figure S2], which is a limitation for using *M. smegmatis* as a model.

In this study, we provided experimental evidence to support the compensatory/resistance effects of several *rpoB* non-RRDR mutations. As genome sequencing has been increasingly applied in clinical settings to predict drug resistance and the fitness of *M. tuberculosis*, our results may help to distinguish between *rpoB* mutations conferring rifampicin resistance and fitness compensation. The compensatory mutations in *rpoB*, as well as those in *rpoA* and *rpoC*, may provide molecular markers to predict highly adapted drug-resistant strains.

## Supplementary Material

Supplementary_materials.doc
