# Supplementary material for: Compensatory effects of M. tuberculosis rpoB mutations outside the rifampicin resistance-determining region
Source: Emerg Microbes Infect. 2021 Mar 29;10(1):743–52. doi: 10.1080/22221751.2021.1908096 (PMC8057087; doi:10.1080/22221751.2021.1908096)

**Supplementary Table S1**. Oligos carrying specific mutations used for screening rifampicin resistance.

| **Oligo** | **Sequence (5′-3′)*** |
| --- | --- |
| Oligo-S450L | CTCACGGGACAGACCGCCGGGGCCCAGCGCCAAAAGACGACGCTTGTGGGTCAGACC |
| Oligo-P45L | GGAATCCGTCTGAACGTCGAGCAGCCCCAGAACCTCAAGCGGTTCACGGAGCTTGGC |
| Oligo-P45S | GGAATCCGTCTGAACGTCGAGCAGCCCCGAAACCTCAAGCGGTTCACGGAGCTTGGC |
| Oligo-V170F | CACGCCAGGCGAACGGACCAGCTGCGAGAACACGACGCGCTCGGTGCCGTTGATGAT |
| Oligo- A286V | GAACAGGTTCTCCAGCAGGGTCTGCACGGACTCCTTGGTCGGCGGCTCGCCCGGGCG |
| Oligo- L378R | CAGGCCCACGCGGATCTGGTTCTGGATGCGCTCACCGACGGTGCGCAGACGACGGTT |
| Oligo- T400A | CTGCGGCGTGATCGCCTCGACGTCCTGGGCGGTCATGCGCTCACGCACGACACGC |
| Oligo- P454L | GCCGGCGCGCTCACGGGACAGACCGCCCAGGCCCAGCGCCGAAAGACGACGCTTGTG |
| Oligo-I480V | GATCAGACCGATGTTGGGACCCTCAGGGGTCTCGACCGGGCACATGCGGCCGTAGTG |
| Oligo-V534M | GTTCTCGTCGGTCGGCGAGTTGGCCTGCGCCACCATGTGGCGGTCCTCCTCGTCGGC |
| Oligo-I491F | CACGCGGGCGTACACCGACAGCGAACCGAACAGACCGATGTTGGGACCCTCAGGGGT |
| Oligo-I491T | CACGCGGGCGTACACCGACAGCGAACCGGTCAGACCGATGTTGGGACCCTCAGGGGT |
| Oligo-I491V | CACGCGGGCGTACACCGACAGCGAACCGACCAGACCGATGTTGGGACCCTCAGGGGT |
| Oligo- F503S | CTTGCGGTACGGCGTCTCGATGAAGCCGGACGGGTTCACGCGGGCGTACACCGACAG |
| Oligo- E563D | GTAGTCCACCTGGTCGGCGGAGACGAAGTCGACCTCGCCGCCCTTCTTGCGGACCAT |
| Oligo-L731P | CACGTCCTCTTCGACCAGGCGGTTCGAGGGGATGATCGCGTCCTCGTAGTTGTGGCC |
| Oligo-E761D | GACGTTCGGGATGTCCCGGGTGATCTCGTCGGCGCCCAGCTTGGTGTCGCGGGCATC |
| Oligo-R827C | ACCGTGCGGGACCTTCAGCGACGTGTCGCAGACCTCGCGCGCCTTCTCACCGAAGAT |
| Oligo-R827L | ACCGTGCGGGACCTTCAGCGACGTGTCGAGGACCTCGCGCGCCTTCTCACCGAAGAT |
| Oligo-H835P | GATGCCGATGACCTTGCCCGACTCACCGGGCGGGACCTTCAGCGACGTGTCGCGGAC |
| Oligo-H835R | GATGCCGATGACCTTGCCCGACTCACCGCGCGGGACCTTCAGCGACGTGTCGCGGAC |
| Oligo-V970M | GAGCTCGCCTTCCTGCGCGCCGTCGAACATCGGGGTCGCCACCGTGCTGTCCGCGGG |
| Oligo^hygS→hygR^ | AATCCCTGTTACTTCTCGACCGTATTGATTCGGATGATTCCTACGCGAGCCTGCGGAA |

* The underscore highlights mutated base.

**Supplementary Table S2.** Primers used for *rpoB* application and sequencing

| **Primers** | **Sequence (5′-3′)** |
| --- | --- |
| 1-F | CCAGAGCAAGTCGAACGCTA |
| 1-R | CCGAGAAATCCTCGATGGGG |
| 2-F | TGATCAACATCCGTCCCGTC |
| 2-R | GTTCGAGAGGATGATCGCGT |
| 3-F  3-R | GGGAAGGCCACAACTACGAG  GGAGATCTTGCGCTTCTGGG |

**Supplementary Table S3.** Primers used for the quantitive real-time PCR assay.

| **Primers** | **Sequence (5′-3′)** |
| --- | --- |
| *amiE*-F | GGTTATTCAGGCATCTTCGCG |
| *amiE*-R | ACGTTCGCGCTCGTTCCAT |
| *sigA*-F | GACATGCAGTGGATCTGCCG |
| *sigA*-R | GTGGCGTAGGTCGAGAACTT |

**Supplementary Table S4.** The list of compensatory mutations previously found in *rpoB* of *M.tuberculosis.*

| **Rifampicin**  **resistance mutation**^a^ | **Secondary non-RRDR mutation**^b^ | **Experiment/** **Predicted by bioinformatics**^c^ | **Organism** | **Reference** |
| --- | --- | --- | --- | --- |
| R448C | D435G | Experiment | *S. enterica* | **[1]** |
|  | P479L |  |  |  |
|  | P483S |  |  |  |
|  | E484A |  |  |  |
|  | R556C |  |  |  |
|  | H593Y |  |  |  |
| S450L | E465K | Experiment | *S. enterica* | **[2]** |
|  | G722A |  |  |  |
|  | Q1056R |  |  |  |
| S450L | V534M | Experiment | BCG | **[3]** |
| S450L | I491V | Predicted by bioinformatics | *M. tuberculosis* | **[4]** |
|  | V496M/L |  |  |  |
|  | H835P/R |  |  |  |

a , b Corresponding position in *M. tuberculosis* β subunit.

^c^ Predicted by bioinformatics listed the mutations that recommended as a high likelihood compensatory mutations.

1. Brandis G, Wrande M, Liljas L, et al. Fitness-compensatory mutations in rifampicin-resistant RNA polymerase. Mol Microbiol. 2012;85(1):142-51.

2. Brandis G, Hughes D. Genetic characterization of compensatory evolution in strains carrying rpoB Ser531Leu, the rifampicin resistance mutation most frequently found in clinical isolates. J Antimicrob Chemother. 2013;68(11):2493-7.

3. Meftahi N, Namouchi A, Mhenni B, et al. Evidence for the critical role of a secondary site rpoB mutation in the compensatory evolution and successful transmission of an MDR tuberculosis outbreak strain. J Antimicrob Chemother. 2016;71(2):324-32.

4. Casali N, Nikolayevskyy V, Balabanova Y, et al. Evolution and transmission of drug-resistant tuberculosis in a Russian population. Nat Genet. 2014;46(3):279-86.

**Supplementary Figure S1.**

**(a)** Screening of RIF-resistance transformants by recombineering. Oligo-S450L as a positive control.


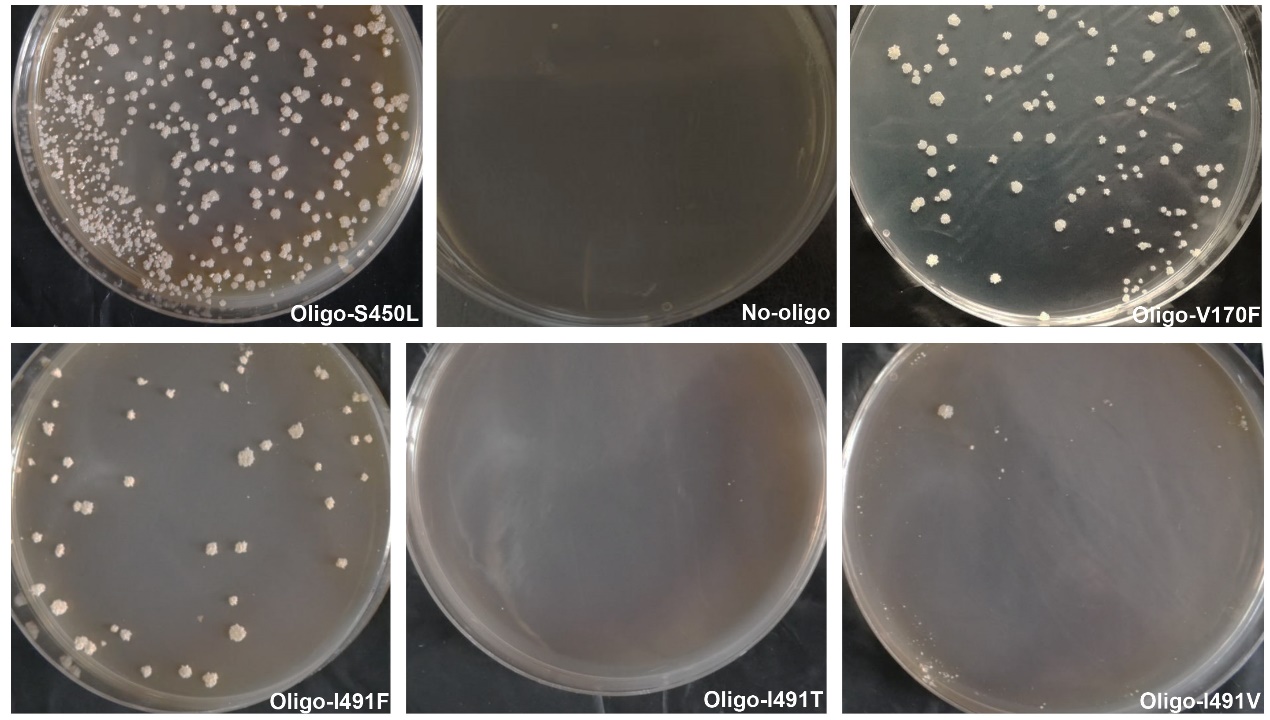


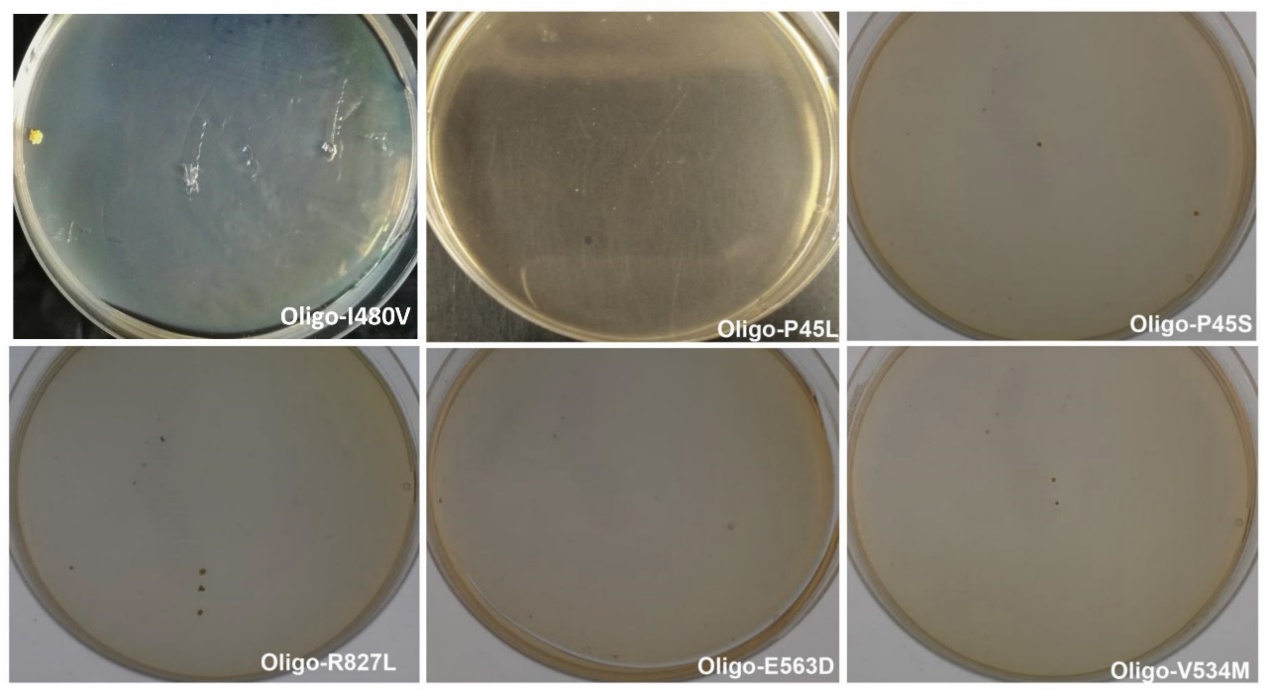


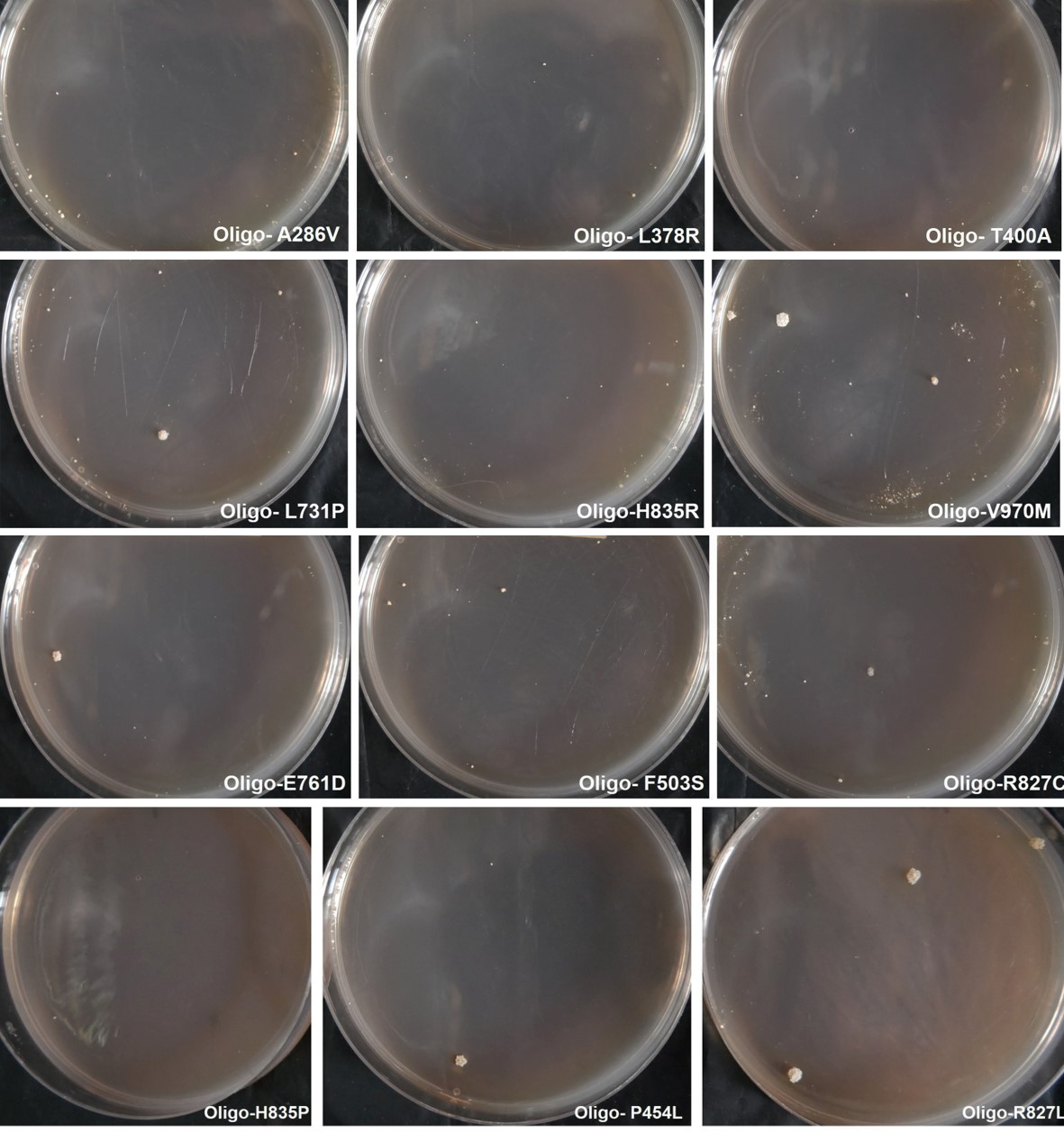


**(b)**. In order to confirm that the transformation and homologous recombination are successful, we co-electroporated the oligos carrying different *rpoB* mutations with the Oligo repairing *hygS* into the Che9c Gp60/61-expressing strain (Oligo^hygS→hygR^), and then, cells were selected on both rifampicin(50μg/ml) and hygromycin(100μg/ml) plates.


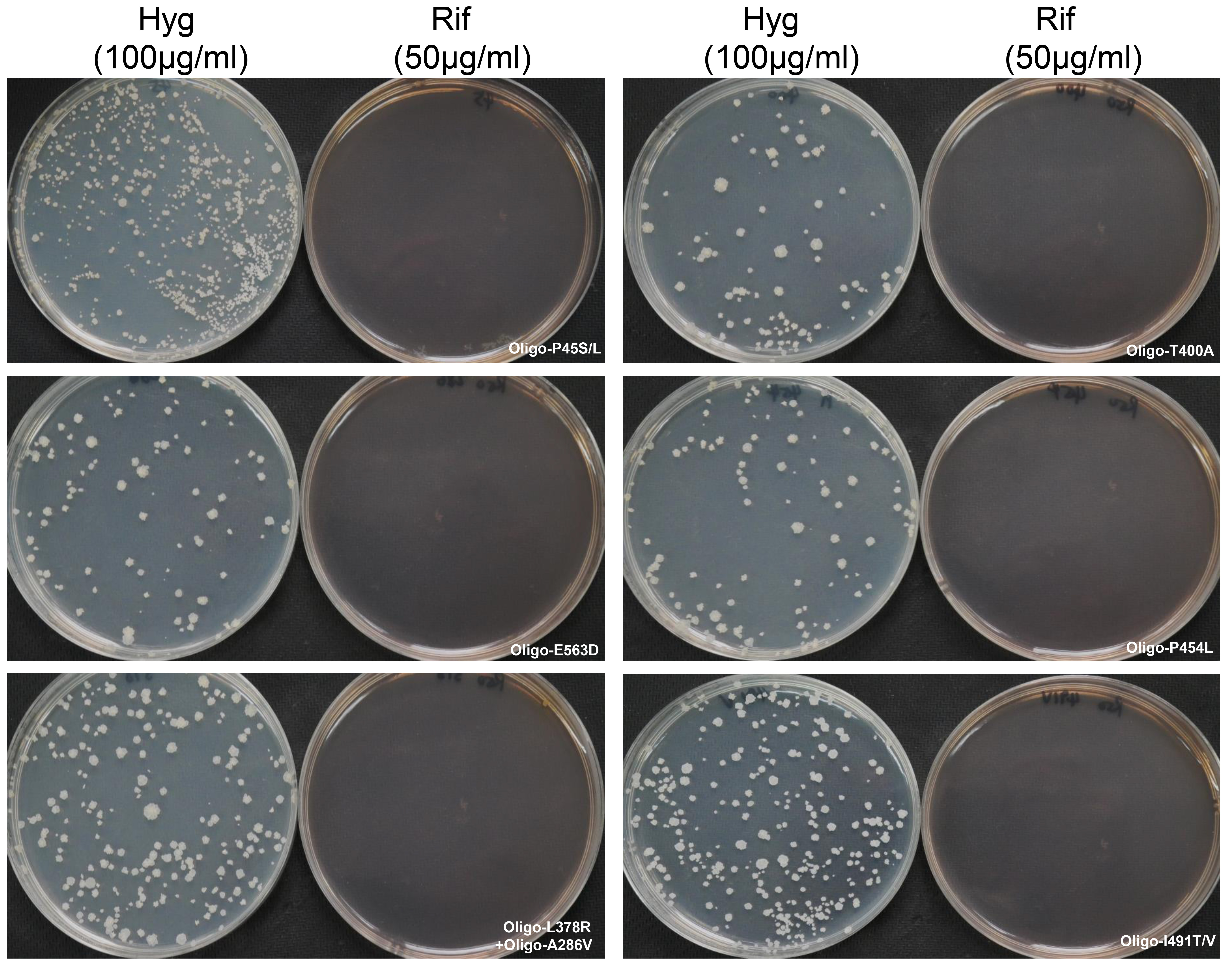


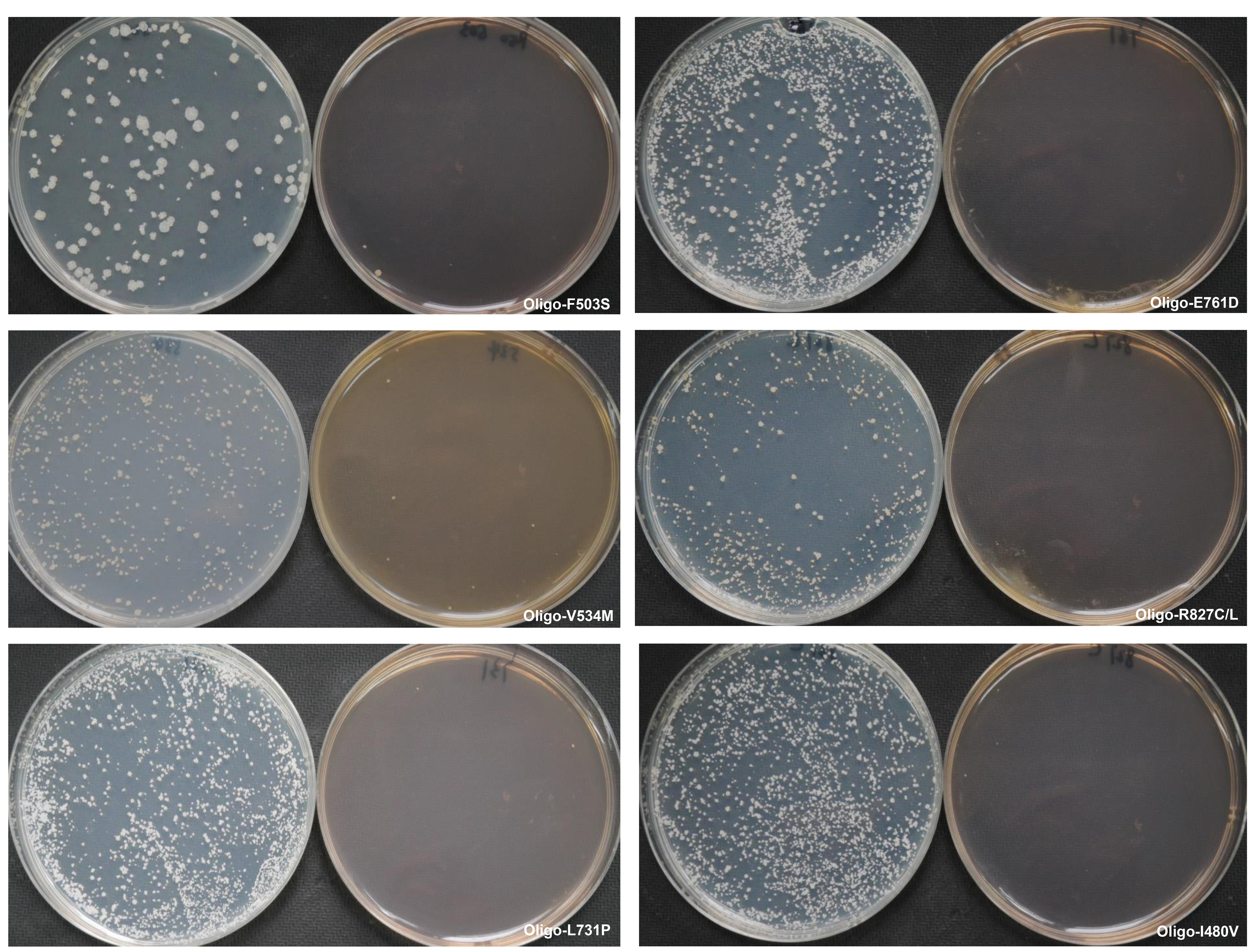


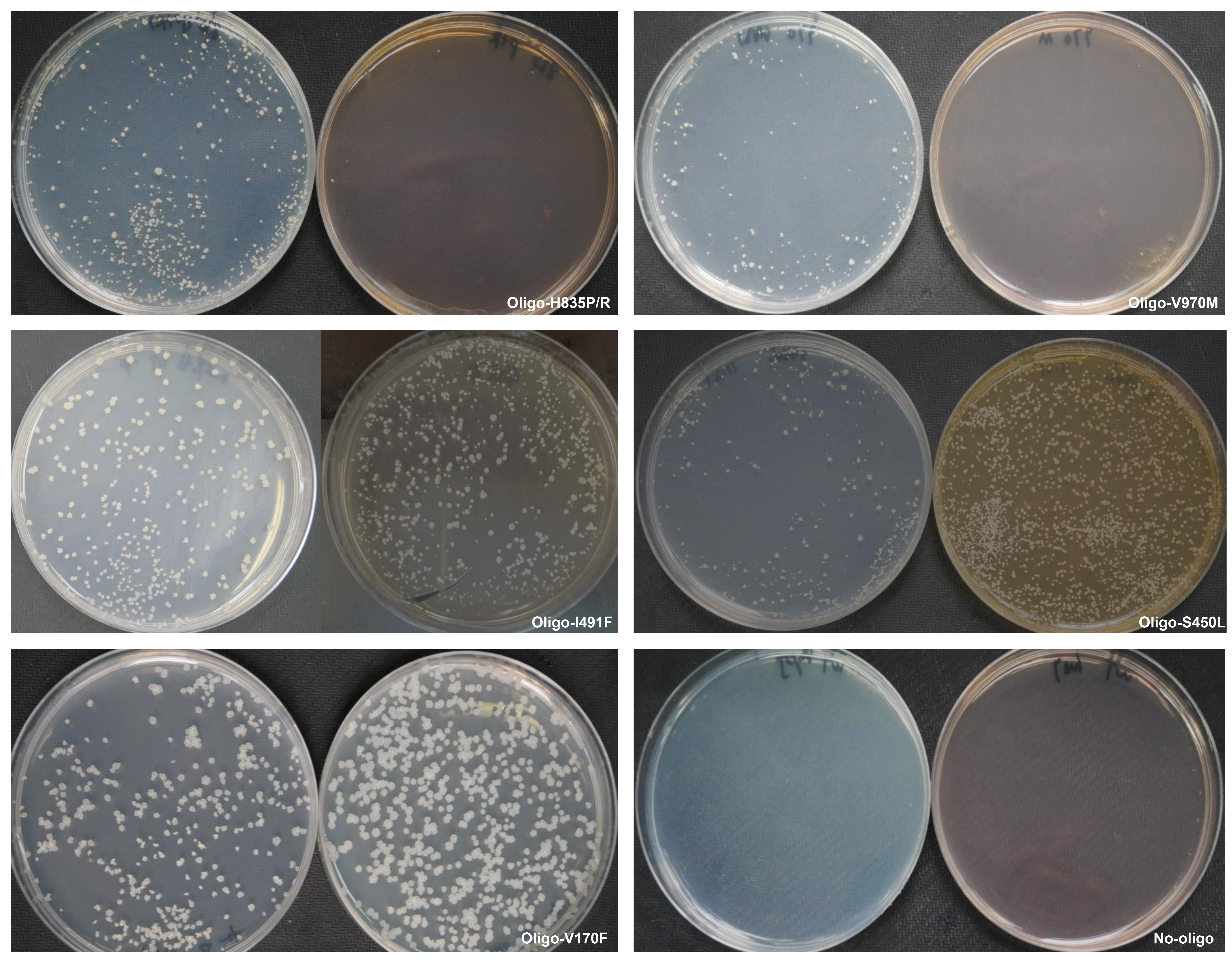


**Supplementary Figure S2.** Protein sequence alignment of RpoB of *M. smegmatis*,

*M. tuberculosis* and *S. enterica.* The RRDR region highlighted with red underscore, green triangles represents compensatory mutations identified in *S. enterica* by Brandis G (J Antimicrob Chemother. 68:2493-7,2013), red triangles represents the mutations that independently emerged at least three times in our analysis.

**Supplementary Figure S3.** The map of pJV53 plasmid constructed in this study. The counter-selectable gene *sacB and* the hygromycin resistance gene (*hygR*) with two adjacent nonsense mutations that inactivate its function were constructed.


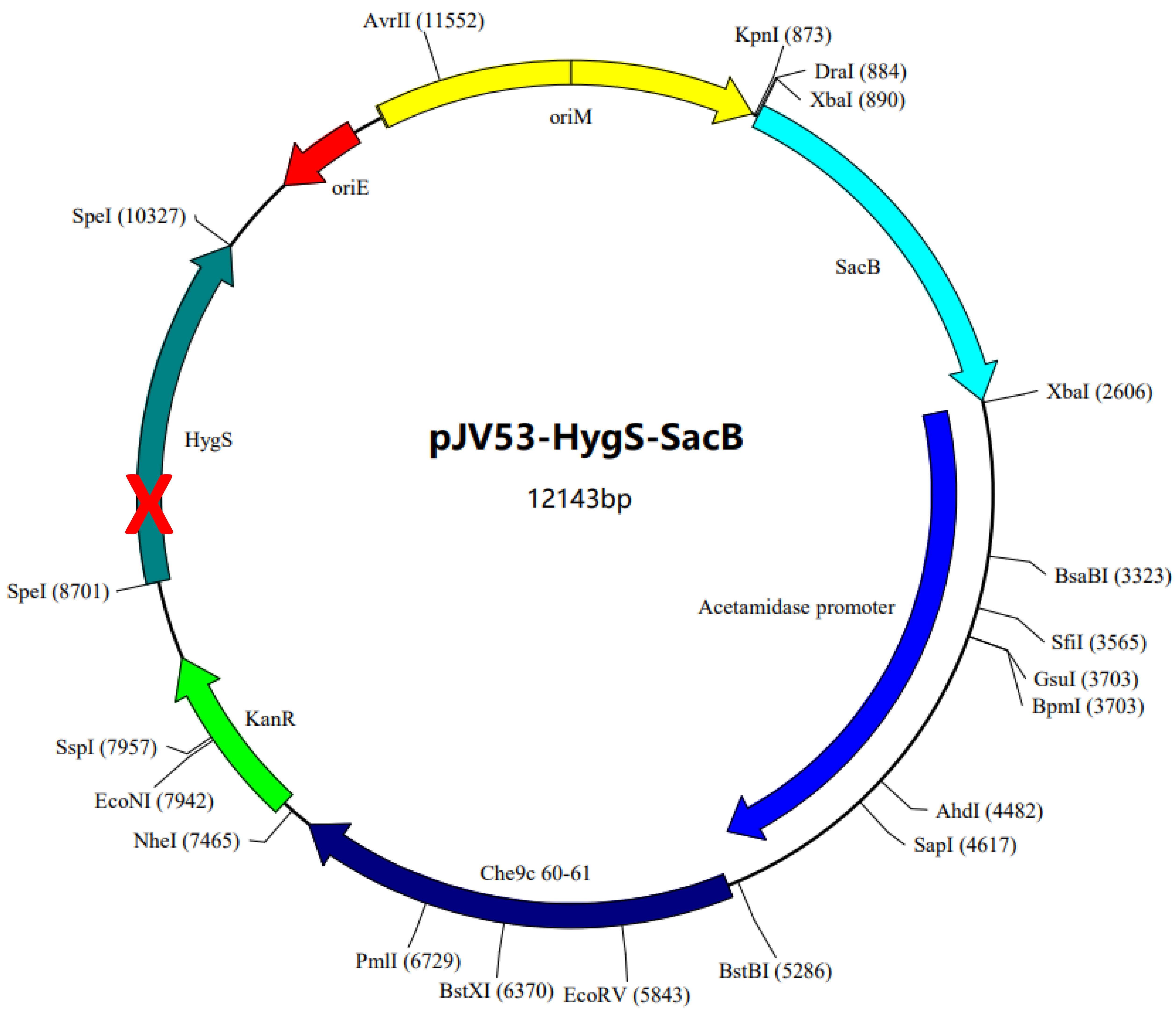

Supplement: Supplementary_materials.doc [file TEMI_A_1908096_SM8041.doc]
